# Use of emergency department services by women victims of violence in Lazio region, Italy

**DOI:** 10.1186/1472-6874-13-31

**Published:** 2013-07-19

**Authors:** Sara Farchi, Arianna Polo, Simona Asole, Maria Pia Ruggieri, Domenico Di Lallo

**Affiliations:** 1 Public Health Agency, Via di Santa Costanza 53, 00198, Rome, Lazio region, Italy; 2 Emergency Department, San Giovanni Addolorata Hospital, Rome, Italy

**Keywords:** Violence, Health services, Women, Public health

## Abstract

**Background:**

Violence against women is a significant health problem and a hidden phenomenon, in Italy that about 31% of the women have been victims of violence once in life. Aims of this study are to describe characteristics of women victims of violence (VV) attending the EDs in the Lazio region in 2008 and to illustrate the frequency and characteristics of previous ED visits.

**Methods:**

Using the Emergency Information System, visits of women, (15–49 years), in the 60 EDs, for a violent trauma have been analysed. For each VV identified, we considered the last episode and searched for ED attendances in a six year period (2003–08) in order to identify other visits. We performed descriptive analyses of socio-demographic and clinical factors of VV and we analyzed the impact previous ED visits. We compared ED utilization of women VV with a random sample of women with the same age distribution who gave birth in 2008.

**Results:**

In 2008, 7,725 ED attendances of women VV were found (1.1% of the ED visits) corresponding to 6,936 women (prevalence = 52.0x10,000). The mean number of ED visits for each woman in five years was 5.0 (1–190). Prevalent diagnoses were contusions (45.8%), neurotic disorders (5.4%) complications of medical care (6.3%). The women were young, approximately 70% were residents in Rome or the surrounding areas. Foreign women were three times more likely to visit the ED for intentional injuries than were Italian women (114.1 vs 44.4 per 10.000).

**Conclusions:**

This study shows high prevalence of violence against women in Lazio region, Italy. Most of the women have been visited by the ED several times before the violent episode, often with traumas. ED medical and nursing staff should be prepared and trained to successfully manage victims of violence.

## Background

Violence is a widespread phenomenon. Each year, more than 1.6 million people worldwide lose their lives to violence. Data from the World Health Organization (WHO) have shown that violence is one of the leading causes of death for people aged 15–44 years worldwide, accounting for about 14% of deaths among males and 7% of deaths among females [1]. In developed countries some studies estimated the percentage of women victims of their spouse or partner to be 25%. The United Nations defines violence against women as “any act of gender-based violence that results in, or is likely to result in, physical, sexual or psychological harm or suffering to women, including threats of such acts, coercion, or arbitrary deprivation of liberty, whether occurring in public or in private life” [2].

A study on 510 women who visited social service or health facilities found high prevalence of violence, mostly perpetrated by men well known to the victims, with 10.2% having experienced physical/sexual violence in the last 12 months, regardless of the perpetrator. Violence by a male partner or former partner occurred to 6.4% of the women; by other relatives, 1.6% of the women; and by “other” persons 3.3% of the women [3]. Other studies confirm that one of the most common forms of violence against women is performed by a husband or an intimate male partner [4-6].

Violence is often unreported and therefore remains a hidden phenomenon. The percentage of women is high that don’t want to speak about their experience (about 34% among victims of intimate partner violence and 24% among victims of violence from unknown persons) [7]. In case of domestic violence most of the episodes are unreported; the reasons are both personal (embarrassment, fear of retaliation, economic dependency) and societal (gender power imbalance in society, family privacy, victim-blaming attitudes) [8]. In case of sexual assault the prevalence estimates are likely underestimated because the event is frequently underreported [9,10]. Only a small proportion of women report the episode. Many women do not report sexual violence to the police because they are ashamed, or fear being blamed, not believed or otherwise mistreated [1].

The WHO in 2002 in the “World Report on Violence and Health” defined steps for the public health approach to violence, where an important step is uncovering as much knowledge as possible about all aspects of violence [1]. As causes and consequences are better known, violence is increasingly becoming a public health priority [11].

An analysis of Emergency Department (ED) visits is one of the instruments useful to quantify and explore the phenomenon because ED is often the first and sometimes the only contact that women have with health care professionals [12]. Several studies have shown that violence can be associated with a variety of health problems [13,14], drug abuse [13], sexual, gynecological [15-17] and gastrointestinal disturbances [18-20]. For these reasons women victims of violence are common visitors to emergency departments [3] and it becomes even more important that health care professionals have skills to recognize hidden signs of violence, such as frequent use of the ED, for example. Rates of domestic violence detection in EDs are still low despite the fact that a high percentage of female victims of domestic violence visit emergency rooms for treatment [21].

Most of the studies collecting data from emergency departments included cases only from one hospital or selected for a survey. There are few studies which estimate prevalence or incidence of the phenomenon using population-based data.

Healthcare professionals are often not prepared to manage these cases either in a human or a professional sense, in fact those who truly understand the “problem of violence”, in the Accident and Emergency units (A&E) and the ED are a minority. A survey carried out by National Statistics Research Institute in Italy confirms that violence cases are ten times more common than those reported by the ED; 69% of ED medical staff report never having suspected a case of domestic violence in their professional experience [7].

A recent study aimed at evaluating the preparedness of the A&Es and ED medical and nursing staff in the Lazio Region showed a lack of technical, professional and psychological training to successfully manage victims of violence, even after years of work experience [22].

The aim of this population-based study is to quantify and describe characteristics of women victims of violence (VV) attending all the EDs in the Lazio region, Italy, in 2008. In order to identify indicators of suspected violence and to detect probably new cases, we retrospectively studied previous ED visits by the same women in a five-year period, and we described the frequency and characteristics of these visits.

## Methods

### Data source

For this study we used the Emergency Information System (EIS) which collects data from all Emergency Department (ED) admissions in the Lazio region (pop = 5,3 million, incl. Rome). It reports: identification code, age and place of birth of the patient; the triage code-urgency scale used to establish treatment priority, up to four diagnoses and up to four therapeutic procedures based on ICD-9-CM; and the final disposition of the ED admission. In case of trauma patients it reports whether or not it is intentional, the place of the unintentional injury, and if the violence was self-inflicted.

**Figure 1:**
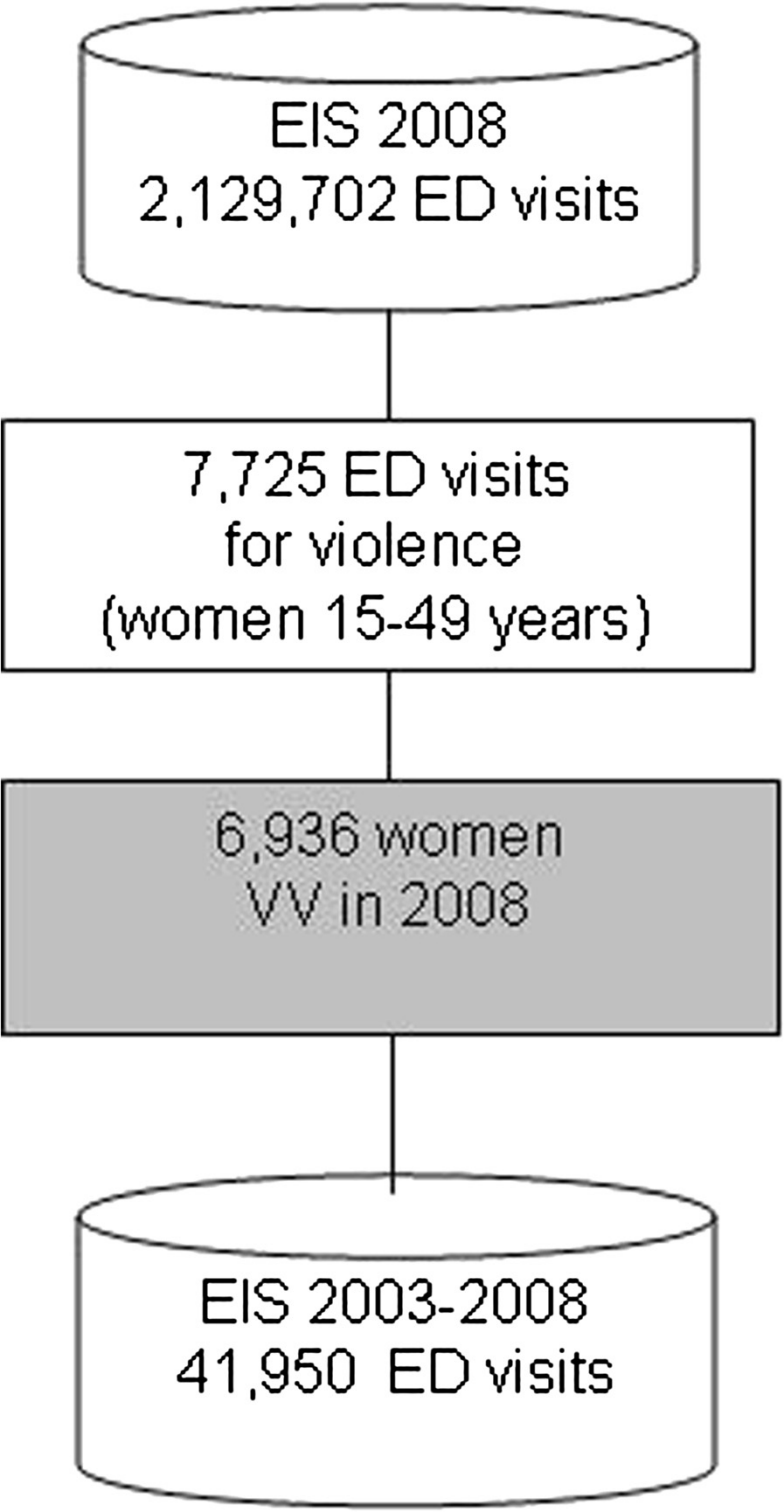
**Selection and retrospective follow up of women victim of violence**. Lazio region, Italy 2003-2008.

In order to identify female VV, we selected the ED visits by women aged 15–49 years in 2008 who claimed to have suffered an “intentional” injury. We also selected ED visits by women whose discharge diagnosis or procedure codes were physical or sexual abuse (ICD9CM codes: 995.80-995.85 or 995.5 or V715 or V716). Through a record linkage procedure we identified the last ED visit caused by intentional injury, called it the “index visit”, then, in order to retrospectively follow up this group of women we performed another record linkage procedure using EIS archives from 2003 to 2008, and selected all the ED visits of the cohort (Figure 1).

In order to compare the number of ED visits of women VV with those done by a control group, we selected from birth registry in 2008 all women aged 15–49 who gave birth in our region. As we have done in case of women VV, we retrospectively extracted all the ED visits done by this population using the personal ID. We then excluded from this group, women with at least one ED visit in the six-year period caused by an intentional injury or who have been abused. Afterward we extracted from this population a random sample with the same age distribution as females VV.

We present socio demographic characteristics of women VV and of the control group and we describe ED utilization by these groups. We also present diagnoses from the ED visit of the VV.

Public Health Agency is a regional authority responsible of managing and analysing administrative health records of Lazio region, in order to perform epidemiological and evaluation studies. We did not ask for ethic committee approval because we performed an observational study, using anonymous data, without performing any treatment on human subjects; results are shown in aggregate form and identification of individuals is not possible.

The management of these data for public health purposes does not require a patient’s informed consent. Data management is performed in respect with the requirements of the current privacy laws in Italy.

## Results

In 2008 we selected from the EIS 7,725 ED visits for intentional injuries the 6,936 women aged 15–49. This population generated 41,950 ED visits in the period 2003–2008. From the archive of the control population we randomly selected 6,936 women of the same age; the number of the ED visits for the control random sample was 30,566. Table 1 reports their socio-demographic characteristics of the VV and of the control women. The women VV were young, 53.6% were under 35 years of age (9% of whom were teenagers), approximately 70% of the VV were residents in Rome or the surrounding areas, 5.7% in the northern and 17.2% in southern areas of the region. Control women showed the same geographical distribution. Most women VV were Italian citizens (76.3%), although foreign women were overrepresented in the VV group compared with control women. Foreign women VV were three times more likely to visit the ED for intentional injuries than were Italian women (114.1 vs 44.4 per 10.000 resident women of the same age). Prevalence was equal to 51.9 per 10.000 women of the same age and resident in the region; it reached a peak in young women aged 20–24 (67.4 per 10,000 women), then it decreased with age. Table 2 shows for each woman in the VV and in the control group the number of previous ED visits. In the VV group 1426 women (20.6%) visited the ED only one time (the index visit) compared with 1171 women (16.9%) in the control group. The mean number of ED visits in six years was 5.0 (1–190), for each woman VV, while it was 3.4 (range: 1–40) for each woman in the control group. The vast majority of women VV visited the ED several times before the index visit with several different diagnoses (with intentional injuries, with unintentional injuries, with other causes), 20.4% visited the ED at least nine times during the six-year period. If we assume that women visited by the ED once in a six-year period, were new cases of violence, incidence would be equal to 10.7 per 10.000 (data not shown). Also the control group visited the ED frequently but not as the VV women.

**Table 1:** Socio-demographic characteristics of female VV, Lazio 2008

	***Women VV***			***Control group***	
	**N**	**%**	**prevalence (per 10.000)**	**N**	**%**
**Age**					
15-19	613	8.8	46.7	613	8.8
20-24	925	13.3	67.4	925	13.3
25-34	2183	31.5	58.0	2183	31.5
35-49	3215	46.4	46.6	3215	46.4
**Residence**					
Rome	3271	47.5	51.5	3420	49.5
Surronding Areas	1523	22.1	45.1	1623	23.5
Northern area	390	5.7	36.2	389	5.6
Southern area	1182	17.2	46.8	1157	16.7
Other region	525	7.6		319	4.6
Total	6891			6908	
Missing	45			28	
**Citizenship**					
Italian	5290	76.3	44.5	5623	81.1
Foreigner	1645	23.7	114.1	1313	18.9
Missing	1	0.0		0	0.0
**Total**	**6936**	**100.0**	**52.0**	**6936**	**100.0**

**Table 2:** History of ED visits of women in the cohort

**N. of previous ED visits (regardless of the reason for the visit) per woman**	***Women VV***		***Control group***	
	**N.**	**%**	**N.**	**%**
1	1426	20.6	1171	16.9
2	1026	14.8	1248	18.0
3	818	11.8	1166	16.8
4	673	9.7	884	12.7
5	478	6.9	645	9.3
6	451	6.5	491	7.1
7	358	5.2	365	5.3
8	290	4.2	234	3.4
9 or more	1416	20.4	732	10.6
**Total**	**6936**	**100.0**	**6936**	**100.0**
**Mean *(min-max) *number of ED visits**	5.0 *(1–190)*		3.4 *(1–40)*	

2003–2008 Lazio region, Italy.

Table 3 shows the ranking of the first fifteen diagnoses assigned by the ED only for the VV group, stratified into three categories: intentional injuries, accidental injuries, and non trauma-related visits. In case of intentional injuries prevalent diagnoses were contusions (45.8%), neurotic disorders (5.4%) complications of medical care (6.3%). There were 351 ED visits classified as sexual abuse (0.7% of all ED visits of the cohort). Diagnoses assigned to accidental injury visits before the index visit are very similar to those assigned as a result of violence. Diagnoses from visits prior to the index visit that do not refer to injury concern symptoms of the abdomen and pelvis (9.1%), complications of medical care (8.0%), anxiety, dissociative and somatoform disorders (4.8%), general symptoms and pregnancy (4.0% each), symptoms related to the respiratory tract (4.3%), menstrual and genital tract disorders (3.0%), and other problems mainly related to pregnancy or gynecological problems, urinary and respiratory system disorders.

**Table 3:** Ranking of the first 15 diagnoses assigned at the ED to women in the cohort during the study period

**ED visits for intentional injury**			**Other unintentional injuries ED visits**			**Non trauma-related ED visits**		
**ICD-9-CM description**	**N**	**%**	**ICD-9-CM description**	**N**	**%**	**ICD-9-CM description**	**N**	**%**
Contusion of face, scalp, and neck except eye	1639	17.17	Contusion of lower limb and of other and unspecified sites	916	13.34	Other symptoms involving abdomen and pelvis	2463	9.65
Contusion of upper limb	1143	11.97	Contusion of upper limb	781	11.37	Complications of medical care, not elsewhere classified	2026	7.93
Contusion of lower limb and of other and unspecified sites	977	10.23	Sprains and strains of other and unspecified parts of back	665	9.68	Anxiety, dissociative and somatoform disorders	1242	4.86
Complications of medical care, not elsewhere classified	648	6.79	Other disorders of cervical region	425	6.19	Normal pregnancy	1228	4.81
Contusion of trunk	557	5.83	Complications of medical care, not elsewhere classified	356	5.18	General symptoms	981	3.84
Anxiety, dissociative and somatoform disorders	481	5.04	Contusion of face, scalp, and neck except eye	354	5.15	Symptoms involving respiratory system and other chest symptoms	605	2.37
Concussion	345	3.61	Sprains and strains of ankle and foot	323	4.70	Symptoms involving urinary system	575	2.25
Sprains and strains of other and unspecified parts of back	315	3.30	Contusion of trunk	272	3.96	Other and unspecified disorders of back	519	2.03
Other disorders of cervical region	294	3.08	Open wound of finger(s)	161	2.34	Hemorrhage in early pregnancy	509	1.99
Other open wound of head	249	2.61	Injury, other and unspecified	137	1.99	Disorders of menstruation and other abnormal bleeding from female genital tract	499	1.95
Fracture of face bones	191	2.00	Sprains and strains of knee and leg	107	1.56	Disorders of conjunctiva	383	1.50
Other disorders of soft tissues	115	1.20	Other open wound of head	96	1.40	Other indications for care or intervention related to labor and delivery, not elsewhere classified	362	1.42
Late effects of injuries to skin and subcutaneous tissues	107	1.12	Sprains and strains of wrist and hand	95	1.38	Cystitis	357	1.40
Contusion of eye and adnexa	107	1.12	Other and unspecified disorders of back	77	1.12	Early or threatened labor	337	1.32

2003–2008, Lazio region, Italy.

We found that among women VV, the 23% of the ED visits were caused by intentional injury, the 16.4% by unintentional injury and the 61% by other clinical causes, while among women included in the control sample the 91% of the ED visits were to by attributed to other clinical causes, mainly related to their condition (pregnancy, labor, delivery), while the remaining 9% were caused by unintentional injuries (data not shown).

## Discussion

To our knowledge, this is one of the first population-based study on Violence against women carried out in 60 ED departments of a wide regional area. Our analysis reveals high prevalence of women visiting the ED because they are victims of violence. Eighty percent of them visited the ED more than once before the index visit for other unintentional injuries and for other reasons. New cases during the study period represent 10.7 per 10.000 inhabitants. We compared the number of ED visits of women VV with a those of a sample of “healthy” women and we found that women VV visited the ED more frequently. If we assume that the control sample was selected because women in it were all pregnant in 2008, and for this reason we assume that they needed health checks by the hospital and the ED compared with the general population, we can suppose that the difference in ED utilization among women VV and general population would be even larger.

Our study has some limitations. It is possible we overestimated prevalence and incidence because we could include only the resident population in the denominator, while it is possible that ED patients may be women temporarily present, in Italy or illegal immigrants. Nonetheless, if we suppose that all the non-resident women are all clandestine and we recalculate the prevalence including only resident foreign women in the numerator we find that prevalence is significantly higher in this group than in Italian women.

Another limitation of this study is the use of ICD 9 CM codes for the identification of cases, which could not correctly classify cases of violence. However, in order to ascertain the validity of this method we used a record linkage procedure to observe the previous history of women. It seems that we effectively we discovered a group of women at high risk of repeat ED utilization.

We are not able to distinguish domestic violence from other forms of violence and we know nothing about the dynamics of the event, we also do not know the patient’s marital status because the information is not present in our archive.

In our study ED visits for intentional injuries are usually classified as “non urgent”, in case of sexual abuse shows that non-urgent triage code was assigned in 71.2% of these visits, indicating that poor attention is given to this phenomenon by the ED staff.

The prevalence of ED visits for violent episodes in women is higher among young and among foreign women. Risk factors of violence victims as reported in the literature are: youth, being pregnant or with young children, being separated or divorced, or lack of stable employment [23,24]. Other risk factors are a history of childhood violence, and alcohol and drug abuse in woman or partner [25].

We used a retrospective approach to consider the history of injury from violence and to identify diagnoses for “suspected violence”.

We found that 80% of our cohort has repeatedly visited the ED, and that diagnoses attributed to previous ED visits are similar to those attributed to violence in case of “unintentional” injuries. Diagnoses assigned to previous ED visits are of disorders found in literature to be frequent in abused women. There’s a body of evidence that suggests that women with a history of violence have poorer overall health and short and long term outcomes [13-20]. We found that diagnoses prior to the index visit were often related to gastrointestinal, genital, pregnancy disorders or to anxiety.

Another study showed that there are fewer women with physical injuries who visit the ED care than those who seek medical care for consequent to violence disorders as anxiety attacks, pain and depression [21].

For all these reasons one would expect female VV to be frequent visitors to emergency departments [3] In our study in a six-year period the average number of ED visits is 5.0. Another study in the US on intimate partner violence cases found that in a three-year period the median number of visits was four [26]. In our study we found that mean number of ED visits increases with time. We can assume that some of these victims try to find help from health service several times before reporting the fact. The response of health services to victims is sometimes to deny the problem [12]. Often battered women in the presence of a physician deny that the injury is intentional and explain them as the result of “a fall down the stairs”, and this version of events is accepted [27]. Healthcare professionals should be trained for early recognition and support of victims of violence, especially considering that the violence is associated to a variety of health problems. The use of protocols for identifying and treating victims of abuse has been found to increase the identification of victims by physicians [28].

Sometimes medical and nursing staff who work in A&Es and EDs lack the technical, professional and social/psychological training to successfully manage victims of violence. This scarcity in training together with a work context where too often protocols for the management of victims of violence are nonexistent - protocols which should be established, shared and implemented by the senior managers and chief physicians of the facilities [8-10]. This situation is unfortunately widespread at A&Es and EDs both within the city of Rome and its suburbs. Furthermore, in spite of the methodology of the Lazio Triage Model (LTM) [Gruppo “Triage Lazio” e Laziosanità 2007], which was designed to protect possible and probable victims of violence, by assigning red or yellow codes to ensure priority of treatment, regardless of whether the violence is overt or suspected and independent of whether their vital functions have been compromised, the triage nurses tend to underestimate these patients, as our results clearly show. This is proof of the enormous scarcity in sensibility and attention to this problem from the health care professionals themselves and the health authorities more generally.

## Conclusions

Our study identified a vulnerable group of the population that requires particular attention from health care services. ED is important, as it is where these women seek care and it can play a relevant role in prevention and detection of cases. The use of ED protocols for the identification and the treatment of the victims can decrease the number of hidden cases.

In the view of the authors, and supported by national and international experience reported in the research literature, this situation could improve through the constitution of a group of experts, doctors and nurses, working in the field of emergency treatment in the Lazio Region, who in collaboration with the relevant institutions, in particular the Agency Public Health, should create: a) a unique protocol for the management of victims of abuse to be implemented in all A&Es and EDs in the region; b) training courses, in collaboration with voluntary organizations and experts in the field, for all staff working in critical areas with periodical re-training; c) a register of victims (data collection for active monitoring and quality control); d) a network of assistance for victims after hospital discharge to be organized in collaboration with NGOs against violence in the Lazio region.

## Competing interests

The authors declare that they have no competing interests

## Authors’ contributions

SF developed the concept, coordinated the study, participated in its design and analysis, helped to draft the manuscript. AP participated in the design and analysis. SA helped to draft the manuscript, MPR helped in the analysis and to draft the manuscript, DDL provided substantial methodological comments on the drafts, contributed to the conception of the research question, assisted in revising the manuscript. All authors reviewed and approved the final manuscript.

## Pre-publication history

The pre-publication history for this paper can be accessed here:

http://www.biomedcentral.com/1472-6874/13/31/prepub

## References

[B1] Krug EG Dahlberg LL Mercy JA Zwi AB Lozano R World report on violence and health 2002 Geneva: WHO

[B2] United Nations General Assembly The United Nations Declaration on the Elimination of Violence against Women 1993 New York: http://www.un.org/documents/ga/res/48/a48r104.htm

[B3] Romito P Gerin D Asking patients about violence: a survey of 510 women attending social and health services in Trieste Italy 2002 54 12 1813 1824 10.1016/S0277-9536(01)00149-6 12113437

[B4] Heise L Garcia-Moreno C WHO Krug EG, Dahlberg LL, Mercy JA, Zwi AB, Lozano R Violence by intimate partners Authors 2002 Geneva: WHO. World report on violence and health

[B5] Novello AC Rosenberg M Saltzman L From the Surgeon General, U.S. Public Health Service JAMA 1992 267 3132 10.1001/jama.1992.03480230024006 1593724

[B6] Garcia-Moreno C Jansen HA Ellsberg M Prevalence of intimate partner violence: findings from the WHO multi-country study on women’s health and domestic violence Lancet 2006 368 9543 1260 1269 10.1016/S0140-6736(06)69523-8 17027732

[B7] ISTAT La violenza e i maltrattamenti contro le donne dentro e fuori la famiglia. Anno 2006 Roma: ISTAT

[B8] Gracia E Unreported cases of domestic violence against women: towards an epidemiology of social silence, Tolerance, and inhibition J Epidemiol Community Health 2004 58 7 536 537 10.1136/jech.2003.019604 15194711 PMC1732820

[B9] Kogan SM Disclosing unwanted sexual experiences: results from a national sample of adolescent women Child Abuse Negl 2004 28 2 147 165 10.1016/j.chiabu.2003.09.014 15003399

[B10] Priebe G Svedin CG Child sexual abuse is largely hidden from the adult society: an epidemiological study of adolescents’ disclosures Child Abuse Negl 2008 32 12 1095 1108 10.1016/j.chiabu.2008.04.001 19038448

[B11] Rutherford A Zwi AB Grove NJ **Violence: a priority for public health? (part 2)** J Epidemiol Community Health 2007 61 9 764 770 10.1136/jech.2006.049072 17699529 PMC2659998

[B12] Gibbons J Services for adult who have experienced child sexual assault: Improving agency response Soc Sci Med 1996 43 12 1755 1763 10.1016/S0277-9536(96)00070-6 8961419

[B13] Richardson J Feder G Domestic violence: a hidden problem for general practice Br J Gen Pract 1996 46 405 239 242 8703527 PMC1239608

[B14] Romito P Molzan Turan J De Marchi M The impact of current and past interpersonal violence on women’s mental health Soc Sci Med 2005 60 8 1717 1727 10.1016/j.socscimed.2004.08.026 15686804

[B15] Weaver TL Impact of rape on female sexuality: review of selected literature Clin Obstet Gynecol 2009 52 4 702 711 10.1097/GRF.0b013e3181bf4bfb 20393422

[B16] Golding J Sexual assault history and women’s reproductive and sexual health Psychol Women Q 1996 20 101 121 10.1111/j.1471-6402.1996.tb00667.x 12296010

[B17] Jamieson DJ Steege JF The association of sexual abuse with pelvic pain complaints in a primary care population Am J Obstet Gynecol 1997 177 6 1408 1412 10.1016/S0002-9378(97)70083-8 9423743

[B18] Baccini F Pallotta N Calabrese E Prevalence of sexual and physical abuse and its relationship with symptom manifestations in patients with chronic organic and functional gastrointestinal disorders Dig Liver Dis 2003 35 4 256 261 10.1016/S1590-8658(03)00075-6 12801037

[B19] Leserman J Drossman DA Relationship of abuse history to functional gastrointestinal disorders and symptoms: some possible mediating mechanisms Trauma Violence Abuse 2007 8 3 331 343 10.1177/1524838007303240 17596349

[B20] Paras ML Murad MH Chen LP Sexual abuse and lifetime diagnosis of somatic disorders: a systematic review and meta-analysis JAMA 2009 302 5 550 561 10.1001/jama.2009.1091 19654389

[B21] Abbott J Johnson R Koziol-McLain J Domestic violence against women: incidence and prevalence in an emergency department population JAMA 1995 273 1763 1767 10.1001/jama.1995.03520460045033 7769770

[B22] Ruggieri MP Guzzo AS Agnanti M Victims of violence in Accident & Emergency: Reporting Survey of Eleven Emergency Structures Out of Eighteen Em Care J 2011 2 VII 13 15

[B23] Romito P De Marchi M Turan JM Identifying Violence Among Women Patients Attending Family Practices: The Role of Research in Community Change J 2004 14 250 265 10.1002/casp.781

[B24] Richardson J Coid J Petruckevitch A Identifying domestic violence: cross sectional study in primary care BMJ 2002 2 7332 274 11823360 10.1136/bmj.324.7332.274 PMC65060

[B25] Wright J Kariya A Characteristics of female victims of assault attending a Scottish accident and emergency department J Accid Emerg Med 1997 14 6 375 378 10.1136/emj.14.6.375 9413777 PMC1342978

[B26] Kothari CL Rhodes KV Missed opportunities: emergency department visits by police-identified victims of intimate partner violence Ann Emerg Med 2006 47 2 190 199 10.1016/j.annemergmed.2005.10.016 16431233

[B27] Romito P Sherr L, St Lawrence J The response of health and social services to battered women Private violence, public complicity 2000 New York: Wiley: Women, health and the mind

[B28] McLeer S Anwar R A study of battered women presenting in an emergency department Am J Public Health 1989 79 65 66 10.2105/AJPH.79.1.65 2909183 PMC1349471

